# Evolutionary Patterns of RNA-Based Duplication in Non-Mammalian Chordates

**DOI:** 10.1371/journal.pone.0021466

**Published:** 2011-07-11

**Authors:** Ming Chen, Ming Zou, Beide Fu, Xin Li, Maria D. Vibranovski, Xiaoni Gan, Dengqiang Wang, Wen Wang, Manyuan Long, Shunping He

**Affiliations:** 1 Key Laboratory of Aquatic Biodiversity and Conservation of Chinese Academy of Sciences, Institute of Hydrobiology, Chinese Academy of Sciences, Wuhan, People's Republic of China; 2 Kunming Institute of Zoology, Chinese Academy of Sciences, Kunming, Yunnan Province, People's Republic of China; 3 Department of Ecology and Evolution, The University of Chicago, Chicago, Illinois, United States of America; 4 Graduate University of Chinese Academy of Sciences, Beijing, People's Republic of China; 5 Yangtze River Fisheries Research Institute, Chinese Academy of Fisheries Sciences, Wuhan, People's Republic of China; Oregon State University, United States of America

## Abstract

The role of RNA-based duplication, or retroposition, in the evolution of new gene functions in mammals, plants, and *Drosophila* has been widely reported. However, little is known about RNA-based duplication in non-mammalian chordates. In this study, we screened ten non-mammalian chordate genomes for retrocopies and investigated their evolutionary patterns. We identified numerous retrocopies in these species. Examination of the age distribution of these retrocopies revealed no burst of young retrocopies in ancient chordate species. Upon comparing these non-mammalian chordate species to the mammalian species, we observed that a larger fraction of the non-mammalian retrocopies was under strong evolutionary constraints than mammalian retrocopies are, as evidenced by signals of purifying selection and expression profiles. For the Western clawed frog, Medaka, and Sea squirt, many retrogenes have evolved gonad and brain expression patterns, similar to what was observed in human. Testing of retrogene movement in the Medaka genome, where the nascent sex chrosomes have been well assembled, did not reveal any significant gene movement. Taken together, our analyses demonstrate that RNA-based duplication generates many functional genes and can make a significant contribution to the evolution of non-mammalian genomes.

## Introduction

RNA-based duplication is a molecular process in which RNA is reverse-transcribed into cDNA and inserted at a new position in the genome. The newly created “retrocopy” usually contains the untranslated and coding regions of the parental gene but does not carry a promoter. It has three alternative evolutionary fates: (i) it may recruit a new regulatory sequence, thus likely acquiring a new expression pattern and forming a new expressed duplicate copy, or “retrogene”; (ii) it may occasionally recruit a regulatory sequence and a new coding region from the insertion site to be translated into a chimeric protein; (iii) it may, more often, lose its coding potential, become a pseudogene, and eventually disappear from the genome. It has been shown that most mammalian retrocopies have become “retropseudogenes” [1]–[4]. However, it has long been expected that retrocopies will be shown to play a significant role in evolution [5]. Many functional retrogenes have been reported in mammals, birds, and invertebrates [1], [3], [6]–[10]. It seems that there are very few RNA-based duplicates in the chicken genome [11]. The reverse transcriptases of the CR1 elements present in chicken have been found to be responsible for the deficiency of retrocopies in that genome [12]–[14]. In contrast, in *Drosophila melanogaster*, about 100 candidate retrogenes have been identified [15]–[17].

Two features characterize the retrogenes of mammals and *Drosophila*. They often show the “expressed in testis” [2] and “out of the X” patterns [3], [15]. Numerous studies [1], [2], [15], [16], [18] have revealed a bias toward retrogene expression in the testis. For example, one study [2] showed that the proportion of testis ESTs that map to retrocopies is higher than that of multi-exon genes, and that a higher proportion of intact retrocopies is expressed in the testis when compared to retropseudogenes. These observations revealed that retrogenes are often transcribed and functional in the testis. In the “out of the X” pattern, a disproportionately large number of retrogenes are derived from parental genes on the X chromosome [2]–[4], [15]. These autosomal retrogenes compensate for the silencing of parental X-linked genes during and after male meiotic sex chromosome inactivation [4]. This out-of-X gene traffic cannot be explained by mutation bias and was driven by natural selection to facilitate male germline function [3].

Chordates (phylum *Chordata*) are a broad class of animals that have in common a notochord with a hollow dorsal nerve cord [19]. The phylum *Chordata* consists of three subphyla *Urochordata*, *Cephalochordata*, and *Craniata*. Subphylum *Urochordata* is represented by the tunicates and *Cephalochordata* by the lancelets. *Craniata* includes the Vertebrata, which in turn includes cyclostomes, fish, amphibians, reptiles, birds, and mammals. Retrogene origination by RNA-based duplication has been reported and analyzed only in mammals, and little is known about retroposition in non-mammalian chordates [20]. To assess the generality of retrocopies (or retrogenes) in non-mammalian chordates, including the distribution and evolutionary patterns, we identified retrocopies (or retrogenes) in ten non-mammalian chordate species. These species included five fish species: the zebrafish (*Danio rerio*), Medaka (*Oryzias latipes*), stickleback (*Gasterosteus aculeatus*), fugu (*Takifugu rubripes*), and *Tetraodon* (*Tetraodon nigroviridis*); one amphibian: the Western clawed frog (*Xenopus tropicalis*); one bird: the chicken (*Gallus gallus*); one reptile: the lizard (*Anolis carolinensis*); one *Urochordate*: the Sea squirt (*Ciona intestinalis*); and one *Cephalochordate*: amphioxus (*Branchiostoma floridae*). Two mammals, human (*Homo sapiens*) and platypus (*Ornithorhynchus anatinus*), were used for comparison. After conducting a systemic evolutionary analysis, we discovered distinct patterns associated with the evolution of retrocopies (or retrogenes) in these non-mammalian chordate species.

## Results

### Distribution of retrocopies in various chordate genomes

**Figure 1:**
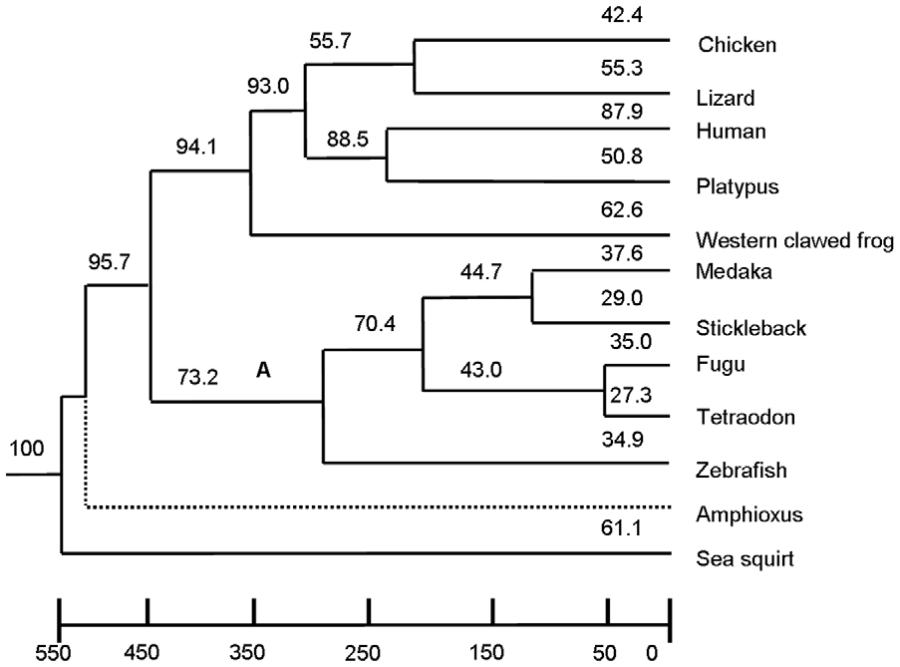
Percentages of LSPs of retrocopies in different species. The percentage of LSPs in a particular lineage (shown above each branch) is the ratio that the number of lineage-specific parent families (LSPs) in the lineage account for the total numbers of parent families the lineage has. Branch A is the lineage Teleostei.

We identified retrocopies in 12 chordate species (phylogenetic relationships are shown in Figure 1) by using the modified computational pipelines in earlier studies [1]. We classified these as either intact retrocopy or retropseudogene according to whether or not they contained frameshift mutations or premature stop codons when compared with their parental genes. In Amphioxus, we found a relatively large number of retrocopies (337), considering the small genome size of this species (Table 1). In the Sea squirt genome and five fish genomes, we identified relatively fewer retrocopies than in non-mammalian tetrapods (other than chicken) such as lizard and Western clawed frog (Table 1). However, the number of retrocopies in lizard and Western clawed frog is lower than that of human and platypus, where 4738 and 542 retrocopies were found.

**Table 1:** Identification of retrocopies in 12 species of Chordata.

*Species*	*Retrocopies number*	*Total protein number*	*P* a *(%)*	*Intact retrocopies*	*Retro-pseudogenes*	*P* b *(%)*	*Retrogene number*	*P* c *(%)*	Genome size (Mb)
Amphioxus	337	50817	0.6%	235	102	70%	176	52%	520
Sea squirt	110	19858	0.6%	96	14	87%	96	87%	173
Zebrafish	195	31743	0.6%	151	44	77%	119	61%	1527
Tetraodon	90	23118	0.4%	66	24	73%	60	67%	342
Fugu	182	47841	0.4%	148	34	81%	142	78%	393
Medaka	218	24661	0.8%	159	59	73%	131	60%	700
Stickleback	132	27576	0.5%	119	13	90%	111	84%	447
Western clawed frog	398	27711	1.4%	216	182	54%	140	35%	1511
Lizard	404	17732	2.2%	217	187	54%	136	34%	1770
Chicken	78	22194	0.4%	57	21	73%	51	65%	1051
Platypus	542	26836	2.0%	146	396	27%	92	17%	1918
Human	4738	47509	10%	565	4173	12%	131	3%	3253

a Percentage of retrocopies per protein.

b Percentage of intact retrocopies among the total retrocopies.

c Percentage of retrogenes among the total retrocopies.

### Higher proportions of the retrocopies were found to be functional in non-mammalian chordates

To deduce retrocopy functionality, we first compared the fraction of intact retrocopies between non-mammalian chordates and mammals. In non-mammalian chordates, the proportion of intact retrocopies ranged from 54% to 87%, significantly (one-tailed Fisher's exact test; *p*<0.01) higher than the proportion of intact retrocopies in the two mammalian species studied here (Table 1), suggesting that a higher percentage of retrocopies are likely to be functional in non-mammalian chordates than in human or platypus.

**Figure 2:**
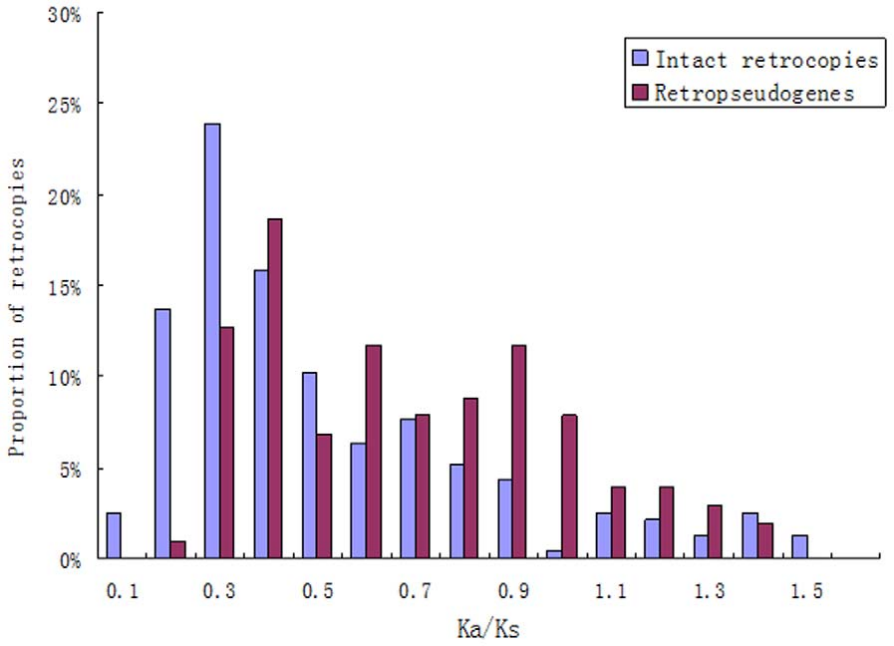
Ka/Ks distributions for intact retrocopies and retropseudogenes in Amphioxus. The Ka/Ks values were obtained through comparing retrocopies and corresponding parental genes.

Secondly, we calculated the ratios of the nonsynonymous substitutions to the synonymous substitutions per site (Ka/Ks) between each retrocopy and its parental gene. Intact retrocopies had different Ka/Ks distributions than retropseudogenes: a higher proportion of intact retrocopies had Ka/Ks<0.5 relative to the proportion of retropseudogenes (one-tailed Fisher's exact test, Table 2). In other words, intact retrocopies were found to be more likely to be under functional constraints. For example, 66% of the intact retrocopies and only 39% of the retropseudogenes had Ka/Ks<0.5 in Amphioxus (Figure 2). There were 27% more intact retrocopies than retropseudogenes were observed with Ka/Ks<0.5. Also, if we defined intact retrocopies with Ka/Ks significantly smaller than 0.5 (see Materials and Methods) as functional retrogenes, only 3% and 17% (Table 1) of retrocopies could be considered to be functional retrogenes in human and platypus, respectively. These proportions are much less than that the 34% to 87% figure for non-mammalian chordates. Moreover, for Sea squirt, stickleback and zebrafish, the total estimated number of retrogenes was only a little smaller than that for human. In amphioxus, fugu, Medaka, Western clawed frog and Lizard, the estimated number of retrogenes was even larger than that for human (Table 1).

**Table 2:** Higher fraction of the retrocopies may be functional in 8 nonmammalian chordates.

*Species*	*Ka/Ks<0.5* a	*Intact and Ka/Ks<0.5*	*Pseudo and Ka/Ks<0.5*	*Fisher's exact test* b	*EST support*	*Intact with EST support*	*Pseudo with EST support*	Fisher's exact test^c^
Western clawed frog	188	117	71	<0.01	150	109	41	<0.01
Zebrafish	97	82	15	0.01	90	82	8	<0.01
Sea squirt	65	60	5	0.05	98	92	6	<0.01
Amphioxus	195	155	40	<0.01	-	-	-	-
Medaka	136	111	25	<0.01	86	79	7	<0.01
Chick	47	42	5	<0.01	-	-	-	-
Fugu	141	121	20	0.01	-	-	-	-
Lizard	248	151	97	<0.01	73	63	10	<0.01

a calculated by using an LPB method.

b Excess of intact retrocopies with Ka/Ks<0.5 relative to retropseudogenes.

c Excess of expressed intact retrocopies relative to retropseudogenes.

Thirdly, for those species that have sufficient expression data, we studied retrocopy expression in them. In Western clawed frog, Sea squirt, zebrafish, stickleback, and Medaka, more than 40% of the retrocopies were expressed, whereas in human, only 27% of retrocopies were expressed. Furthermore, there was a significant excess of expressed intact retrocopies relative to expressed retropseudogenes in these five genomes (one-tailed Fisher's exact test, *p*<0.01, Table 2). This suggests that intact retrocopies were more likely to be expressed than retropseudogenes. Taken together, this evidence suggests that a larger fraction of the retrocopies is likely to be functional in the eight non-mammalian chordates studied (Table 2) than in the two mammals studied.

### Retrogene expression in the gonads and brains of non-mammalian chordates

We analyzed the EST information (http://genome.ucsc.edu/) of seven species under study and summarized the relevant statistics in Table 3. Given the total number of ESTs, the human genome expresses a relatively small proportion of its retrocopies (27%), whereas Medaka, stickleback, zebrafish, and Western clawed frog express about 40% or more of their retrocopies, even though fewer total EST sequences are available than for human (Table 3). At the extreme, 89% of the retrocopies in Sea squirt are transcribed. Only 18% of the retrocopies in the lizard genome appeared to be expressed perhaps because there is much less total expression information available (Table 3). Except in human, most of the expressed retrocopies were found to be intact.

**Table 3:** Total EST analysis of retrocopies.

*Species*	*Number of EST*	*Number (e)* a	*Intact (e)*	*Pseudo (e)*	Percentage (%)^b^
Lizard	156802	73	63	10	18%
Sea squirt	1213772	98	92	6	89%
Medaka	666358	86	79	7	39%
Stickleback	279365	56	54	2	42%
Zebrafish	1511074	90	82	8	46%
Human	9217591	1268	342	926	27%
Western clawed frog	1290068	150	109	41	38%

a These data are of expressed (e) retrocopies.

b The percentage of expressed retrocopies in the total retrocopies of each.

We further analyzed the tissue distributions of the expressed retrogenes (Table 4). In most of the species under study, many functional retrogenes were expressed in the brain. In Western clawed frog, lizard, Medaka, zebrafish and Sea squirt, many functional retrogenes were expressed in the testis or ovary. We explored whether retrogenes were expressed more often in the brain and gonad than in other tissues. Table 4 shows statistics suggesting that this is true in the human, Western clawed frog, Medaka, and Sea squirt genomes.

**Table 4:** Tissue distribution of functional expressed retrogenes.

*Species*	*Tissue*	*N* a *(%)*	*Tissue*	*N (%)*	*Tissue*	*N (%)*	*Tissue*	*N (%)*	*Tissue*	*N (%)*	*Test1* b	Test2^c^
Sea squirt	blood cells	54.9	gonad	45.1	digestive gland	31.9	heart	18.9	neural complex	17.6	p<0.05	-
Medaka	brain	28.0	testis	22.7	ovary	21.3	liver	10.7	eye	2.7	p<0.01	p<0.01
Stickleback	brain	63.6	gills	36.3	eyes	29.1	skin	12.7			-	NA
Zebrafish	heart	12.3	gills	9.6%	testis	8.2	ovary	8.2	brain	8.2	-	-
Lizard	testes	41.7	brain	25.0	ovary	22.2%	Regenerating tail	19.4	Dewlap	13.9	NA	NA
Western clawed frog	brain	43.9	testis	41.5	Liver	14.6	Lung	14.6	Intestine	9.8	p<0.05	p<0.05
Human	testis	58.3	brain	55.0	hippocampus	33.3%	placenta	26.7	Melanotic melanoma	21.7	p<0.01	p<0.01

a percentage of expressed retrogenes in every tissue among total expressed retrogenes.

b test whether there are more retrogenes expressed in gonad, binary logistic regression.

c test whether there are more retrogenes expressed in brain.

### Gene traffic in the Medaka genome

In this study, we tested the “out of the X” hypothesis in the non-mammalian chordate genomes. The sex-determining system of Medaka is XX–XY [21], but the differentiation of the sex chromosomes seems to be in an early stage. Chromosome 1 acts as the X chromosome, whereas the Y chromosome is a variant form of chromosome 1 with a 250-kb Y-specific region that contains the male-determining gene, DMY [22]. This suggests an early stage in the evolution of sex chromosomes [23]. We identified 131 functional retrogenes in the Medaka genome. Of these, five genes were from the sex chromosome. About 3.6 autosomal retrogenes were expected from the X chromosome, which is not significantly different from the observed value (five, Fisher's exact test, two-tail, *p* = 0.75), revealing no excess of autosomal functional retrogenes from the X chromosome in Medaka.

### Age distribution of retrocopies

**Figure 3:**
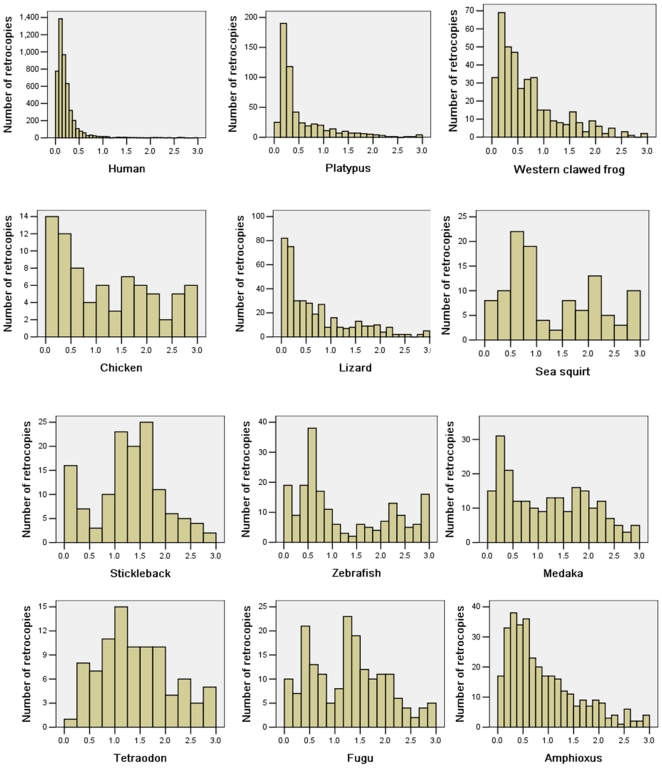
Ks distribution of retrocopies in 12 chordates. The Ks values were obtained through comparing retrocopies and corresponding parental genes.

Figure 3 shows the Ks distribution of retrocopies in all these species. It also shows that, for tetrapods other than chicken, there are many young retrocopies. However, no burst of young retrocopies has been found in ancient chordates such as Amphioxus, Sea squirt or fish. For example, assuming a neutral mutation rate of 1–1.3×10^−9^ substitutions per site per year in primates [24], about 1352 retrocopies were generated in the human genome within 38–50 million years. The Western clawed frog, *Xenopus tropicalis*, and the African clawed frog, *X. laevis* diverged about 63.7 million years ago [25]. A Ks value of 0.292 corresponds to the divergence between these two species [26]. There are about 85 retrocopies in the Western clawed frog, which with a Ks<0.23 (0.292×50/63.7), originated within about 50 million years. However, for zebrafish, the divergence of the *Danio rerio* and *Cyprinus carpio* species occurred about 50 million years ago [27]. We compared 38 pairs of orthologous genes [28] between *D. rerio* and *C. carpio* and obtained an overall Ks value of 0.413. Only 32 retrocopies had a Ks<0.413 and originated within 50 million years. For fugu and Tetraodon, the amount of neutral substitution (Ks) since the Tetraodon–Fugu divergence was 0.35 [29], there are only 18 retrocopies in Fugu originated within the last 50 million years, which is the approximate time of divergence of these two species [28], [29]. Notably, there is only one retrocopy in Tetraodon with Ks<0.35.

### Chimeric retrogenes identified in the Zebrafish and Western clawed frog

**Figure 4:**
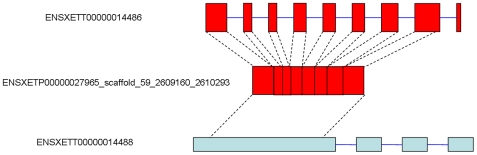
A chimerical retrocopy in Western clawed frog. Red boxes represent exons of parental and retrocopy; light blue boxes represent exons of chimeric gene, and blue lines represent introns.

To identify chimeric retrogenes, we defined Ensembl-annotated genes sharing 30%∼70% of their coding sequences with our retrocopies as a chimeric retrogenes. By this criterion, we found nine chimerical retrogenes in the zebrafish and sixteen in the Western clawed frog (Table 5, for more information, please see supplemental Table S1 and Table S2); 89% and 50% of chimeric coding structures were confirmed by mRNA or EST sequences in zebrafish and Western clawed frog respectively (Table 5). For example, out of nine chimeric retrogenes in zebrafish, seven genes matched at least one mRNA sequence with >98% identity, spanning the whole coding region. One chimeric retrogene matched one EST sequence that spanned both the recruited coding sequence and retrosequence. Figure 4 shows an exemplified chimeric retrogene in the Western clawed frog. The parental gene ENSXETT00000014486 has nine exons. Of these, eight exons were reverse-transcribed and formed a retrocopy. This retrocopy inserted into the first exon of a host gene and formed the chimeric retrogene ENSXETT00000014488.

**Table 5:** Evidence of chimeric coding structure in Zebrafish, Western clawed frog.

*Species*	*Chimeric retrogenes*	*mRNA*	*EST*	*Merged*	Percentage^b^
Zebrafish	9	7^a^	1	8	89%
Western clawed frog	16	7	1	8	50%

a mRNA or EST sequences that span both recruited coding sequence and retrosequence.

b the percentage of chimeric retrogenes with evidence of chimeric coding structure.

### In non-mammalian chordates retrocopies may be mainly produced by LINE1 elements

Retrocopies have been shown to be generated by LINE1 elements in human [30]–[32]. However, it is not known whether retrocopies are mainly produced by LINE1 or other LINE elements in non-mammalian chordates. We used RepeatMasker [33] to identify different kinds of LINE elements in all these species (except Amphioxus and lizard, and the data for human and platypus came from [34] and [35], respectively). We found the number of retrocopies correlated with the number of LINE1 copies (*p*<0.001, Pearson correlation test; Table 6) but not with any other type of LINE element. Furthermore, in the chicken genome, the total number of retroelements was not small, although only 78 retrocopies were detected. We analyzed the LINE elements in the chicken genome, and found most to be CR1 elements, which seemed likely to have generated negligible number of retrocopies [11]. As in the chicken, we found that CR1 elements also dominate the LINE elements of the Western clawed frog genome. In contrast, we found 4074 LINE1-like elements and 398 retrocopies in the Western clawed frog genome. Two pufferfish, fugu and Tetraodon, diverged only 50 million years ago [28], and the number of retrocopies found in fugu was about twice that of Tetraodon, which is consistent with the fact that there are more LINE1 elements in fugu than in Tetraodon.

**Table 6:** The relationships between retrocopy number and the copy numbers of different kinds of LINE elements.

*Species*	*Retrocopies*	*LINE1*	*LINE2*	*CR1*	RTE
Zebrafish	195	4653	54088	0	6105
Western clawed frog	398	4074	0	73281	0
Platypus	572	60	19109700	437600	856900
Medaka	218	698	0	0	29
Human	4738	516000	315000	0	0
Fugu	182	1411	13283	0	4150
Tetraodon	90	324	2043	0	1974
Stickleback	132	16	0	0	1
Sea squirt	110	7597	5007	0	0
Chicken	78	0	10000	205000	0
significance^a^		p<0.001; r = 0.994	NS^b^	NS	NS

a Pearson correlation test; r is correlation coefficient.

b Not Significant.

### Gene family of parental genes

Pan and Zhang [36] recently identified retrofamilies of more than one retrocopy present in only one lineage. These they called “lineage-specific retrofamilies” (LSRs). Because most of the retrocopies that we identified have not been annotated by Ensembl, they were not assigned to any LSRs. However, to investigate the characteristics of the parental genes that generated the retrocopies, we classified them according to the Ensembl gene family annotation, and mapped the percentages of lineage-specific parent families (LSPs) of the retrocopies onto the species tree (Figure 1). We can see terminal branches of branch A, whose divergence times are not as long as those of other branches, as the species listed there have lower proportions of LSPs (27.3%–37.6% in Fugu). On the contrary, the proportion of LSPs is over 40% on all the other branches, increasing to 87.9% in human. This high proportion of LSPs in the human genome results in higher proportions in the related internal branches.

## Discussion

In this study, we identified numerous retrocopies in ten non-mammalian chordate species. We observed obvious differences in the evolution of RNA-based duplication between mammalian and non-mammalian chordates. In mammals, most retrocopies are retropseudogenes [1], [2]. In non-mammalian chordates, most retrocopies are intact. Amphioxus, Sea squirt, two pufferfish, Medaka, and stickleback have small genomes (Table 1), and the retropseudogenes in small genomes may degenerate faster than those of species with large genomes [37], [38]. For example, given that, in pufferfish, the rate of DNA loss per nucleotide substitution is approximately five times faster and the rate of neutral mutation is about 2.5 times faster than in mammals, the retropseudogenes should have degenerated more than ten times faster in the pufferfish genomes than in mammalian genomes [29]. The Ks distribution of retropseudogenes (supplemental Figure S1) also supports this conclusion in that there are rare, old retropseudogenes in these compact genomes. Moreover, in compact genomes, there is usually a stronger selection against deleterious insertions [39]. Only the functional beneficial retrocopies are likely to be retained and fixed. Notably, zebrafish and Western clawed frog have large genomes of about 1.5 Gb, but the fractions of intact retrocopies in these species are also high (above 54% to 77%). Interestingly, the size of the platypus genome is similar to that of the lizard, zebrafish and Western clawed frog (Table 1), but most of the retrocopies in the platypus genome are retropseudogenes (as many as 73%).

The duplicated retrocopies might be a result of “subfunctionalization” [40]. Further analysis indicated that a higher fraction of the retrocopies was likely to be functional in the non-mammalian chordates than the in the two mammals studied, as supported by the analyses of evolutionary constraints and expression profiles. Moreover, the number of functional retrogenes in the eight non-mammalian chordate species (excepting chicken and Tetraodon) was close to the number of functional retrogenes in the human genome, although the total number of retrocopies in these species was found to be an order of magnitude lower than in human.

Retrogenes have evolved some common tissue-biased expression patterns. In general, they are preferentially expressed in the testis, brain and ovary. Previous work has shown that retrogenes tend to be expressed in the testis in both mammals and *Drosophila* [1], [2], [15], [16], [18]. Our research shows that many retrogenes are expressed in the testis not only in human, but also in Western clawed frog, Medaka and Sea squirt. Two hypotheses could explain this observation [14], [39], [41]. The first is that a hypertranscription state exists in meiotic and postmeiotic spermatogenic cells. This state allows the transcription of retrocopies in the testis that would not usually be transcribed. Some retrocopies then acquire a beneficial function and evolve into functional retrogenes. The second is that retrocopies are preferentially inserted into or close to germline-expressed genes. The leaky expression of germline-expressed genes allows some retrogenes to be expressed in the germline [14]. As in the testis, we also found that many retrogenes were expressed in the brain, in accordance with previous observation in primates [1].

In Medaka, the hypothetical “out of the X” movement was not observed. This result is consistent with the fact that the differentiation of the sex chromosomes in Medaka is primitive [42]. These results, in conjunction with previous tests in Populus [43], indicate that “out of the X” patterns are not detectable for the nascent sex chromosomal systems.

Our observations also showed that the number of retrocopies of these chordates correlated with the number of LINE1 copies in these species, suggesting an experimentally testable prediction: that the retrocopies in the non-mammalian chordates may also be mainly produced by LINE1 elements as mammalian retrocopies are.

We identified nine chimerical genes in zebrafish and sixteen chimerical genes in the Western clawed frog. The drastic changes in the protein structures in these genes likely brought up the novel functions, as has been previously observed in the *Drosophila* new gene, *jingwei* [44]. This provides evidence that the non-mammals evolved under positive selection for new gene functionality.

This study identified large numbers of retrogenes in the non-mammalian chordates. Further investigation of these retrogenes revealed some common evolutionary patterns. A similar rate of functional retrogene origination was found throughout the evolution of chordates, in spite of the fact that the processed pseudogenes evolved in diverse rates. Many retrogenes evolved gonad- and brain-based expression patterns. Moreover, we performed an analysis on two non-mammal species, the Western clawed frog and zebrafish, and found sixteen and nine chimerical genes reside in their genomes, respectively. This may suggest that the acquisition of drastically new protein functions accompany the evolution of these chordate organisms.

## Materials and Methods

### Retrocopy identification

To identify retrocopies in the twelve genomes studied (Table 1), we adapted an approach previously used in humans [1]. All genome sequences and annotated protein datasets for these species except those for amphioxus were downloaded from Ensembl (http://www.ensembl.org/). (For zebrafish and Medaka, the data are release 50; humans, release 53; all others, release 52.) The amphioxus genome sequences were obtained from the website of the Joint Genome Institute (http://genome.jgi-psf.org/).

For each species, a TBLASTN [45] analysis was performed using all the protein sequences as queries against the whole-genome sequences. Homologous HSPs (high-scoring segment pairs) were chained together using a dynamic programming algorithm. Homologous chains that had more than 60% alignable regions and more than 40% identity to the query protein were considered homologous genes. Using GeneWise [46], we identified homologous genes without introns (or gaps more than 40 bp) from the exon coordinates as candidate genes.

Next, all the candidate genes were aligned with all the Ensembl proteins using FASTA [47]. We only retained those alignments with >40% identity and an alignment length of at least 40 amino acids. The candidate genes were regarded as candidate retrocopies if the best hit was a gene with multiple coding exons (having introns larger than 70 bp). We then checked whether the introns of the parental gene (the best hit) had been lost or retained in the retrocopies. If introns were retained, the retrocopy we identified may be false-positive and should be discarded. To further reduce the number of false-positive candidates, we removed candidate retrocopies with only one less intron than the parental gene. We also used RepeatMasker to remove all candidates with more than 50% repeat elements. The identified retrocopies were further classified as intact retrocopies or retropseudogenes according to whether their open reading frames were disrupted (by frameshift mutations or premature stop codons) compared with those of the parental genes.

### Ka and Ks estimation and functional retrogenes

The retrocopies were aligned with their parental genes. The Ka and Ks substitution rates and the Ka/Ks ratios were calculated with KaKs_calculator_1.2 [48] using the LPB [49], [50] method. We defined the intact retrocopies with Ka/Ks<0.5 (p<0.01) as functional retrogenes via the *codeml* program in PAML3.14 [51], [52]. This method compares a model in which Ka/Ks is fixed to 0.5 (null model) to a model in which Ka/Ks is estimated from the data. Twice the log likelihood difference was compared to a χ^2^ distribution with one degree of freedom.

### Expression and functional analyses

The expression data were downloaded from the UCSC (http://hgdownload.cse.ucsc.edu/downloads.html). Our retrocopy sequences were then mapped onto them using BLAST. If a retrocopy had an overlap of more than 200 bp and more than 98% identity, we considered it to be expressed. We also downloaded tissue information about the expressed functional retrogenes from NCBI using Batch Entrez (http://www.ncbi.nlm.nih.gov/). We downloaded Ensembl gene family information using BIOMART (http://www.ensembl.org/).

### Chimeric retrogene screen

For the zebrafish and western clawed frog, there were abundant mRNA and EST sequences that could be considered evidence of chimeric structure, so we only identified chimeric retrocopies in these two genomes. After we obtained the retrocopies, we compared the gene position of Ensembl annotated genes to our retrocopies and identified any overlapping pairs. Then we performed a TBLASTN search using these Ensembl annotated genes as queries against overlapped retrocopies and their parental genes. The Ensembl annotated genes with at least 30% coding sequences that not matching the retrocopies or parental genes (with flanking 50,000 bp) were regarded as chimeric retrogenes.

### LINE elements and retrocopies numbers

The LINE elements of the human and the platypus [35] were obtained from published articles, and we performed a repeat analysis of the different chordate genomes using RepeatMasker and the RepBase database [33]. To avoid false-positive LINE1 hits, a Smith–Waterman score of 250 was chosen as the cut-off value.

### Statistics

In this study, we used Fisher's exact test to determine whether an excess of intact retrocopies with Ka/Ks<0.5 or existed or were expressed relative to retropseudogenes. Binary logistic regression was used to determine whether there were more retrogenes expressed in the gonads or brain relative to other tissues. The Pearson correlation test was used to determine whether the number of retrocopies correlated with different kinds of LINE elements. The expected number of retrogenes from the X chromosome was determined according the method described by Vinckenbosch *et al.* [2].

## Supporting Information

Figure S1 (PPT)

Table S1 (XLS)

Table S2 (XLS)
