# Supplementary figures and images for: Evolutionary Patterns of RNA-Based Duplication in Non-Mammalian Chordates
Source: PLoS One. 2011 Jul 11;6(7):e21466. doi: 10.1371/journal.pone.0021466 (PMC3136929; doi:10.1371/journal.pone.0021466)

## Slide 1
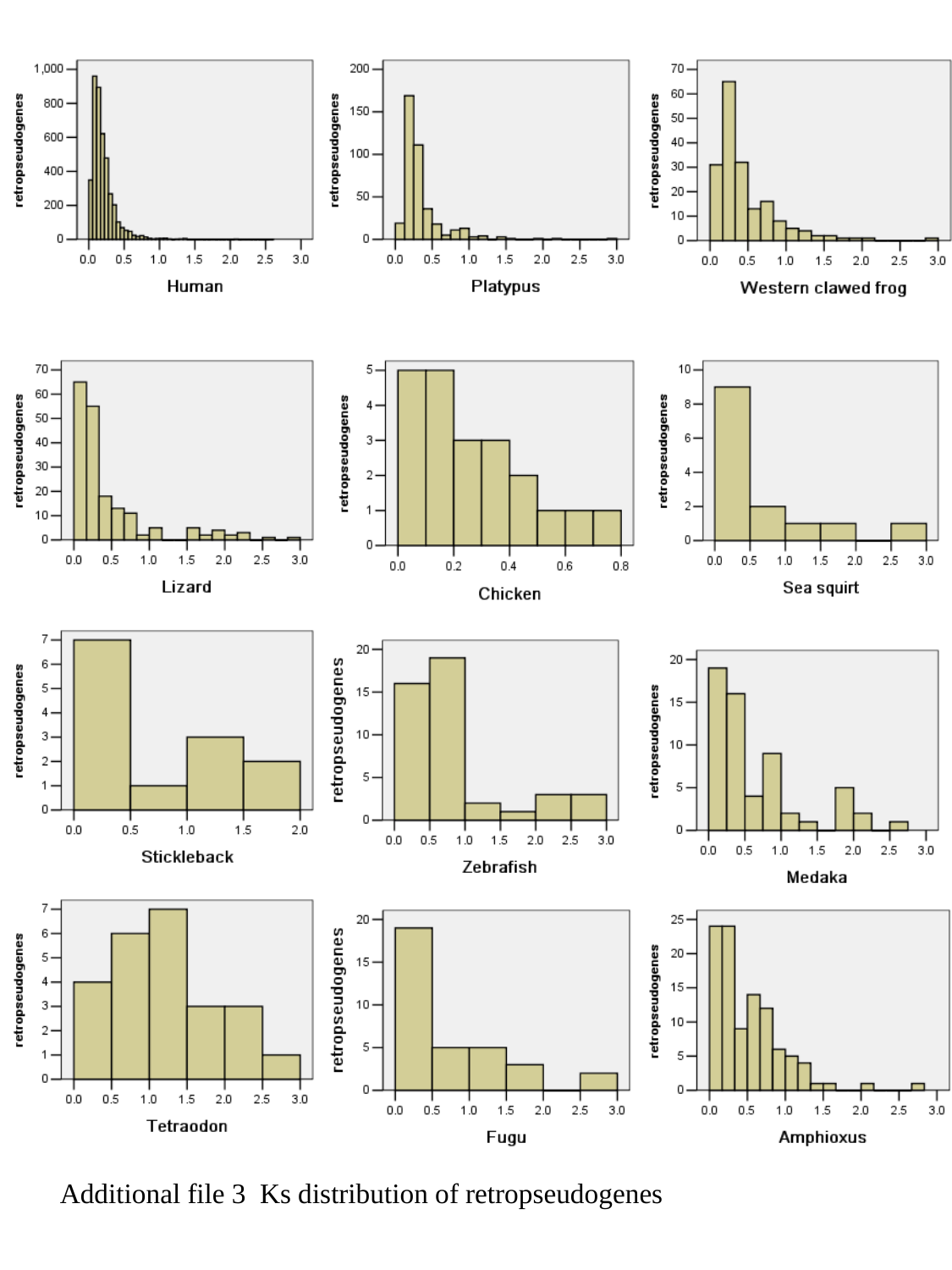

Additional file 3 Ks distribution of retropseudogenes

Supplement: Figure S1 — (PPT) [file pone.0021466.s001.ppt]
